# Psilocybin for major depressive disorder: a systematic review of randomized controlled studies

**DOI:** 10.3389/fpsyt.2024.1416420

**Published:** 2024-09-23

**Authors:** Li-Juan Li, Yu Mo, Zhan-Ming Shi, Xing-Bing Huang, Yu-Ping Ning, Hua-Wang Wu, Xin-Hu Yang, Wei Zheng

**Affiliations:** ^1^ Department of Neurology, The First Affiliated Hospital of Hainan Medical University, Haikou, China; ^2^ Department of Psychology, The Brain Hospital of Guangxi Zhuang Autonomous Region, Liuzhou, China; ^3^ Department of Psychology, Chongqing Jiangbei Mental Health Center, Chongqing, China; ^4^ The Affiliated Brain Hospital, Guangzhou Medical University, Guangzhou, China; ^5^ Key Laboratory of Neurogenetics and Channelopathies of Guangdong Province and the Ministry of Education of China, Guangzhou Medical University, Guangzhou, China

**Keywords:** psilocybin, major depressive disorder, systematic review, efficacy, randomized controlled trial

## Abstract

**Objectives:**

The purpose of this systematic review of randomized controlled trials (RCTs) was to evaluate the effectiveness, safety, and tolerability of psilocybin in adult patients with major depressive disorder (MDD).

**Methods:**

A systematic search (up to September 14, 2023) was conducted for RCTs that examined the efficacy, safety, and tolerability of psilocybin in physically healthy adult patients with MDD. Three independent researchers extracted data from publications where the primary outcome was a change in depressive symptoms, and key secondary outcomes were changes in anxiety symptoms and suicidal ideation, discontinuation rates for any reason, and adverse drug reactions (ADRs).

**Results:**

Five RCTs with 472 adult patients with MDD on psilocybin (n = 274) and controls (n = 198) were included. Two of the five RCTs (40%) reported mixed results, while the other three (60%) found that psilocybin had a beneficial effect on MDD treatment. Four RCTs (80%) assessing the anxiolytic effects of psilocybin for treating MDD found that psilocybin was significantly more effective than the control group in improving anxiety symptoms. Psilocybin was more effective than the control group in improving suicidal ideation in one out of five RCTs. Discontinuation rates were similar for any reason between the psilocybin group (2–13%) and the control group (4–21%) (P > 0.05). Four RCTs (80%) reported ADRs in detail. The most common ADR in both groups was headache.

**Conclusion:**

Psilocybin was effective in improving depressive symptoms in over half of the included studies and reduced anxiety symptoms in patients with MDD. The long-term efficacy and safety of psilocybin for MDD treatment needs to be further investigated in large RCTs.

## 1 Introduction

Major depressive disorder (MDD) is a highly prevalent condition in society (1) and is characterized by severe, persistent, unremitting depression, anhedonia, feelings of powerlessness, and guilt (2). MDD can lead to disability and is associated with an increased risk of mortality (3). The most common pharmacological treatments for MDD are selective serotonin reuptake inhibitors, serotonin and norepinephrine reuptake inhibitors, and other related drugs that selectively target neurotransmitters (4). A meta-analysis of 21 common antidepressant drugs in adults with MDD found that all were more effective than the placebo in improving depressive symptom severity, but the effect sizes were small (5). These drugs have a delayed onset of action, requiring weeks to months of treatment, high side effect rates, high relapse rates, and chronic dosing (6). Therefore, new treatments that are more effective and work faster to improve depressive symptoms are needed.

Novel pharmacological interventions, such as ketamine/esketamine (7) or psilocybin (8), have shown positive results in treating patients with MDD and have the potential to provide better protection. Treatment of depression with single or multiple infusions of ketamine is safe and effective (9, 10). In addition, multiple infusions of ketamine have cumulative and sustained antidepressant effects (9). A recent systematic review found that esketamine and psilocybin were effective in reducing depression symptoms and after overcoming some limitations, could be regarded as possible novel antidepressants (11). Compared to psilocybin, ketamine has a higher potential for addiction and toxic effects (12, 13), such as ulcerative cystitis (14).

Psilocybin is a naturally occurring psychoactive alkaloid that acts as an unselective agonist of many serotonergic receptors, particularly 5-hydroxytryptamine 2A (5-HT_2A_) (15). Studies have increasingly shown that psilocybin can be effective in treating mood disorders and reducing anxiety and depression symptoms (16). However, the findings of RCTs (8, 17–20) of psilocybin examining the efficacy and tolerability of psilocybin in patients with MDD have been inconsistent. Previous systematic reviews and meta-analyses have examined the efficacy and tolerability of psilocybin in patients with MDD. Some of these reviews included both primary and secondary depression (21–23), while others focused only on secondary depression, such as patients with life-threatening cancer (24, 25). For example, a recent meta-analysis found that psychedelics were significantly more effective than a placebo in reducing anxiety and depression among patients with cancer or other life-threatening diseases (24).

To date, no published systematic reviews have examined the efficacy and safety of psilocybin, focusing solely on physically healthy adults with MDD. Thus, we conducted this systematic review to examine the antidepressant, anxiolytic, and anti-suicidal effects and tolerability of psilocybin as an adjunctive treatment for physically healthy patients with primary MDD.

## 2 Methods

### 2.1 Data sources and search strategy

Three investigators, LJL, ZMS, and YM, independently, searched six online databases, including PubMed, Cochrane Library, PsycINFO, EMBASE, Chinese Journal Net, and WanFang databases, from the time the databases were launched until September 14, 2023. The following search terms were used: (“psilocybin” [Mesh] OR psilocybin OR psilocybine OR psilocibin OR psiloc*) AND (“depressive disorder” [Mesh] OR “depression”[Mesh] OR depress* OR dysthymi* OR adjustment disorder* OR mood disorder* OR affective disorder OR affective symptoms) AND (random* OR placebo OR control). The reference lists of the included trials (8, 17–20) and relevant review articles (16, 23, 26, 27) were manually searched to find any additional trials. Although the study protocol was not registered, this systematic review followed the PRISMA guidelines (**Supplementary Table 1**).

### 2.2 Study criteria and data extraction

This systematic review was conducted in accordance with the Preferred Reporting Items for Systematic Reviews and Meta-Analyses (PRISMA) guidelines (Liberati et al., 2009). Studies were selected and assessed for inclusion according to the following ***PICOS*** criteria. ***P*** articipants: Physically healthy patients (≥ 18 years old) diagnosed with MDD according to international diagnostic criteria. ***I*** ntervention versus ***C*** omparison: adjunctive psilocybin (e.g., psychological support plus psilocybin) versus control (e.g., psychological support plus placebo) groups. ***O*** utcomes: The primary outcome was changes in depressive symptoms as measured by standardized scales (e.g., the Montgomery-Åsberg Depression Rating Scale [MADRS] (28), 17-item Hamilton Depression Rating Scale [HAMD-17] (29), Beck Depression Inventory [BDI] (30), 9-item Patient Health Questionnaire [PHQ-9] (31), or 16-item Quick Inventory of Depressive Symptomatology-Self Report [QIDS-SR-16] (32)). Key secondary outcomes were changes in anxiety symptoms as measured by standardized scales (e.g., the Hamilton Anxiety Rating Scale [HAMA] (33), the Spielberger’s Trait Anxiety Inventory [STAI] (34)) and suicidal ideation as measured by standardized scales (e.g., the Colombia-Suicidality Severity Rating Scale [C-SSRS] (35), the Suicidal Ideation Attributes Scale [SIDAS] (36), the Food and Drug Administration Classification Algorithm of Suicide Assessment 2012 [FDA-CASA 2012] (37)), the rates of discontinuation for any reason, and adverse drug reactions (ADRs). ***S*** tudy design: Published single-blind or double-blind RCTs focusing on the efficacy, safety, and tolerability of adjunctive psilocybin in physically healthy patients with MDD. Studies focusing on patients with chronic and serious physical illnesses, such as cancer (38–41), or healthy volunteers (42–46) were excluded. Only studies with the most complete data were included (8, 17, 18) when there were multiple publications based on the same dataset. Additionally, as previously recommended (47), open-label studies (48–51) were excluded. This systematic review did not include any review articles, retrospective studies, or case reports/series.

Three investigators, LJL, ZMS, and YM, independently extracted data from each included study. The three investigators (LJL, ZMS, and YM) discussed any discrepancies in data entry and consulted the senior author (WZ) when necessary. We contacted the first and/or corresponding author to obtain missing information when necessary.

### 2.3 Quality assessment

Three investigators LJL, ZMS, and YM, independently assessed the quality of each included RCT using the Jadad scale (52) and Cochrane Risk of Bias (53). As previously reported (54), RCTs were considered high quality if the Jadad score was ≥ 3.

## 3 Results

### 3.1 Literature search

**Figure 1:**
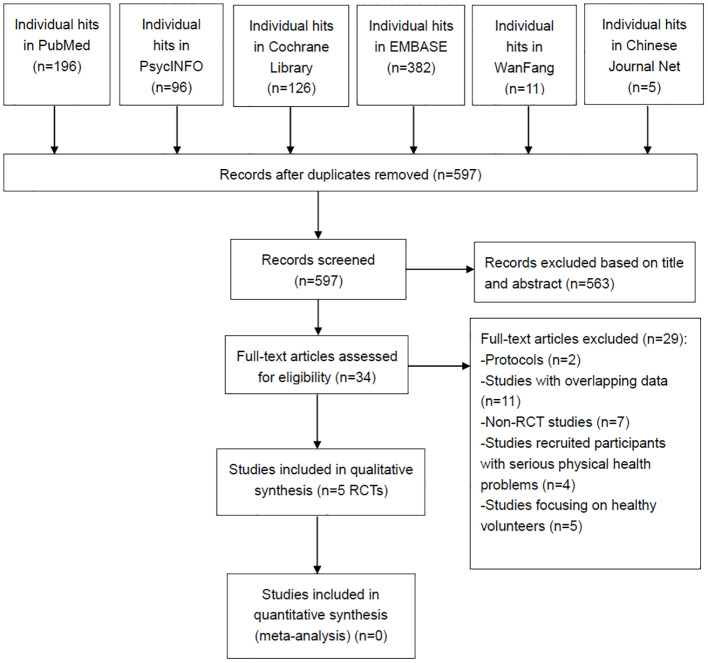
PRISMA flow diagram. PRISMA, Preferred Reporting Items for Systematic Reviews and Meta-analyses; RCTs, randomized controlled trials.

**Figure 1** shows that 816 articles were initially identified from the six databases mentioned above. After removing 219 duplicates, 563 records were excluded based on their title and abstract. Finally, 34 articles were screened for full text. Five RCTs (8, 17–20) met the inclusion and exclusion criteria for this systematic review.

### 3.2 Patient and study characteristics

**Table 1** summarizes the characteristics of each RCT. The study samples had a mean age ranging from 36.8 to 41.2 years, with 48.4% of patients being male. Approximately 16% of the patients had used psilocybin or other psychedelics prior to this study. In two RCTs (8, 17), patients were given two separate doses of psilocybin (session 1: 25mg and session 2: 25 mg in Carhart-Harris et al.’s study (8); session 1: 20 mg/70 kg and session 2: 30 mg/70 kg in Davis et al.’s study (17)). In the remaining RCTs (18–20), patients were given a single dose of psilocybin (0.215 mg/kg or 1–25 mg). The RCTs included in the study varied in duration from 2 to 12 weeks.

**Table 1:** Participant characteristics and psilocybin treatment parameters for each included study.

Studies (country)	N^a^	Design: -Blinding -Setting	Participants: -Criteria (TRD %) -Illness duration (yrs)	Gender^b^: Male (%)	Age^b^: yrs (range)	Previous psilocybin/psychedelic use: n (%)	Invention and control groups; number of participants	Invention: -Sessions -Interval time (wks)	Trial duration (wks)^c^	Jadad score
Carhart-Harris et al., 2021 (UK)	59	-DB -Outpatients	-DSM-IV -MDD (0) -18.7	39 (66.1)	41.2 (21-64)	16 (27.1)	1. Psilocybin (session 1: 25 mg; session 2: 25 mg) + placebo (psilocybin 1 mg) + psychological support^f^; n=30 2. Placebo (psilocybin session 1: 1 mg; session 2: 1 mg) + escitalopram (10 mg; 20 mg) + psychological support^f^; n=29	-2 -3	6	5
Davis et al., 2021 (USA)	27^d^	-SB -Outpatients	-DSM-5 -MDD (NR) -21.5	8 (33.3)	39.8 (21-75)	6 (25.0)	1. Psilocybin (session 1:20 mg/70 kg; session 2: 30 mg/70 kg) + psychological support^g^; n=13 2. Psychological support^g^; n=11	-2 -1.6	8	3
Goodwin et al., 2022 (Multicenter^e^)	233	-DB -Outpatients	-DSM-5 -MDD (100%) -NR	112 (48.1)	39.8 (≥18)	14 (6.0)	1. Psilocybin (25 mg) + psychological support^g^; n=79 2. Psilocybin (10 mg) + psychological support^g^; n=75 3. Psilocybin (1 mg) + psychological support^g^; n=79	-1 -NA	12	5
Raison et al., 2023 (USA)	104	-DB -NR	-DSM-5 -MDD (12.5%) -NR	52 (50.0)	41.1 (21-65)	23 (22.1)	1. Psilocybin (25 mg) + psychological support^g^; n=51 2. Niacin (100 mg) + psychological support^g^; n=53	-1 -NA	6	5
von Rotz et al., 2023 (Switzerland)	52	-DB -Outpatients and inpatients	-DSM-IV -MDD (NR) -NR	19 (36.5)	36.8 (18-60)	16 (30.8)	1. Psilocybin (0.215 mg/kg) + psychological support^g^; n=26 2. Placebo (pure mannitol) + psychological support^g^; n=26	-1 -NA	2	5

a Data were extracted based on random assignment.

b Available data were extracted based on the mean baseline value of each included trials.

c The trial duration was defined as the entire period from begin of the interventions to the assessments of primary and secondary outcomes, while the waiting list trial only extracted first-stage data.

d A total of 27 participants were randomized, of whom 24 completed the intervention as well as the possession assessments at weeks 1 and 4; specifically, 13 were randomized to the immediate treatment group and 11 to the delayed treatment group.

e Including 22 sites in 10 European countries (the Czech Republic, Denmark, Germany, Ireland, the Netherlands, Portugal, Spain, and the United Kingdom) and North America (Canada and the United States).

f Incorporating three components: 1) a sense of emotional support and trust; 2) music listening; and 3) setting a therapeutic intention ahead of a psychedelic experience.

g Nondirective support.

DB, double blind; DSM-IV, Diagnostic and Statistical Manual of Mental Disorders 4th edition; DSM-5, Diagnostic and Statistical Manual of Mental Disorders 5th edition; MDD, major depressive disorder; N, number of patients; NR, not reported; NA, not applicable; SB, single blind; TRD, treatment-resistant depression; wks, weeks; yrs, years.

### 3.3 Quality assessment

**Figure 2:**
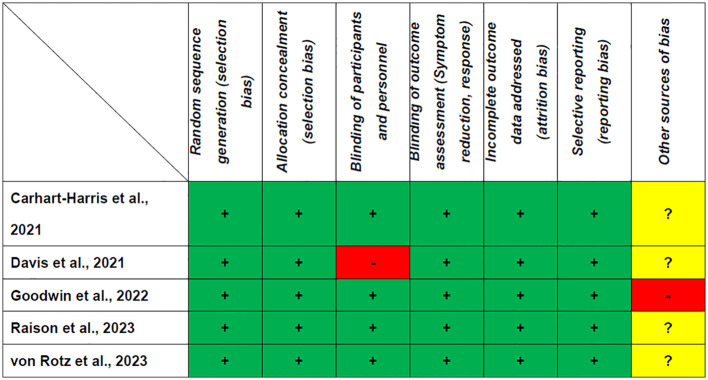
Cochrane risk of bias. +: Low risk of bias; -: High risk of bias; ?: Unclear risk of bias.

**Table 1** shows that all the RCTs included in this study met the criteria for high quality. The Jadad score ranged from 3 (1 RCT) (17) to 5 (4 RCTs) (8, 18–20). As shown in **Figure 2**, all RCTs were assessed as having a low risk of bias for selection, detection, attrition, and reporting.

### 3.4 Clinical efficacy

#### 3.4.1 Changes in depressive symptoms

**Table 2** shows that two RCTs (40%, 2/5) (8, 18) reported mixed findings, while three RCTs (60%, 3/5) (17, 19, 20) found a positive effect of psilocybin in treating MDD. Specifically, one RCT (8) found that psilocybin was more effective than escitalopram in improving depressive symptoms as measured by the BDI, HAMD-17, and MADRS, but not the QIDS-SR-16 at week 6. Another RCT (18) found that a single dose of 25 mg of psilocybin, but not 10 mg, significantly improved depressive symptoms more than a 1 mg dose, as measured by the MADRS on day 2 and weeks 1 and 3. The remaining three RCTs (17, 19, 20) found that psilocybin significantly improved depressive symptoms, as measured by the HAMD-17, MADRS, QIDS-SR-16, PHQ-9, or BDI, and that the effects lasted from 2 to 6 weeks (**Table 2**).

**Table 2:** Psilocybin for depression: clinical efficacy.

Studies	Assessment scales	Findings
The severity of depressive symptoms		
Carhart-Harris et al., 2021 (UK)	BDI, HAMD-17, MADRS and QIDS-SR-16	Psilocybin was more effective than escitalopram at week 6 in improving depressive symptoms as measured by the BDI, HAMD-17, and MADRS (secondary outcomes) but not the QIDS-SR-16 (primary outcome).
Davis et al., 2021 (USA)	BDI, HAMD-17, QIDS-SR-16 and PHQ-9	Psilocybin significantly improved depressive symptoms as measured by the BDI, HAMD-17, QIDS-SR-16, and PHQ-9 at weeks 1 and 4 post-session 2.
Goodwin et al., 2022 (Multicenter)	MADRS	Psilocybin, at a single dose of 25 mg, but not 10 mg, significantly improved depressive symptoms more than a 1 mg dose, as measured by the MADRS at day 2 and weeks 1 and 3. Improvement in depressive symptoms at weeks 6, 9, and 12 between the 25 mg group or the 10 mg group and the 1 mg group, as measured by the MADRS showed no significant differences.
Raison et al., 2023 (USA)	MADRS	Psilocybin was significantly superior to niacin in improving depressive symptoms on the MADRS at weeks 1, 2, 4, and 6.
von Rotz et al., 2023 (Switzerland)	BDI and MADRS	Psilocybin was significantly more effective than a placebo in improving depressive symptoms as measured by the BDI and MADRS at days 0, 2, and weeks 1, 2.
The severity of anxiety symptoms		
Carhart-Harris et al., 2021 (UK)	STAI	Psilocybin was more effective than escitalopram at week 6 in improving anxiety symptoms as measured by the STAI.
Davis et al., 2021 (USA)	HAMA and STAI	Psilocybin significantly improved anxiety symptoms as measured by the HAMA and STAI at week 4 post-session 2.
Goodwin et al., 2022 (Multicenter)	NR	NR
Raison et al., 2023 (USA)	HAMA	Psilocybin was significantly more effective than niacin in improving anxiety symptoms on the HAMA at weeks 1, 2, 4, and 6.
von Rotz et al., 2023 (Switzerland)	HAMA	Psilocybin was significantly more effective than a placebo in improving anxiety symptoms as measured by the HAMA at week 2.
The severity of suicidal ideation		
Carhart-Harris et al., 2021 (UK)	SIDAS	Psilocybin was more effective than escitalopram at week 6 in reducing suicidal ideation as measured by the SIDAS.
Davis et al., 2021 (USA)	C-SSRS	Psilocybin did not significantly reduce suicidal ideation as measured by the C-SSRS.
Goodwin et al., 2022 (Multicenter)	FDA-CASA 2012	Suicidal ideation occurred in all dose groups, and no significant differences were observed between the three dose groups as measured by the FDA-CASA 2012.
Raison et al., 2023 (USA)	C-SSRS	No significant group differences between psilocybin and niacin in reducing suicidal ideation as measured by the C-SSRS.
von Rotz et al., 2023 (Switzerland)	C-SSRS	No significant group differences between psilocybin and placebo in reducing suicidal ideation as measured by the C-SSRS.

BDI, Beck Depression Inventory; C-SSRS, Colombia-Suicidality severity rating scale; FDA-CASA, Food and Drug Administration Classification Algorithm of Suicide Assessment, conversion of C-SSRS and S-STS (Sheehan Suicidality Tracking Scale); HAMD-17, 17-item Hamilton Depression Rating Scale; HAMA, Hamilton Anxiety Rating Scale; MADRS, Montgomery-Åsberg Depression Rating Scale; NR, not reported; NS, not significant; PHQ-9, 9-item Patient Health Questionnaire; QIDS-SR-16, 16-item Quick Inventory of Depressive Symptomatology-Self Report; SIDAS, Suicidal Ideation Attributes Scale; STAI, Spielberger’s Trait Anxiety Inventory.

#### 3.4.2 Changes in anxiety symptoms

Four RCTs (80%) (8, 17, 19, 20) assessed the anxiolytic effects of psilocybin in treating MDD. Among these RCTs, it was found that psilocybin was significantly better than the control group at improving anxiety symptoms, as measured by the HAMA and/or STAI (**Table 2**).

#### 3.4.3 Changes in suicidal ideation

All included RCTs (8, 17–20) assessed the anti-suicidal effects of psilocybin in treating MDD. Only one RCT (20%) (8) found that psilocybin was more effective than escitalopram in reducing suicidal ideation as measured by the SIDAS, while the other four RCTs (80%) (17–20) found that psilocybin did not significantly reduce suicidal ideation as measured by the C-SSRS (three RCTs) (17, 19, 20) and FDA-CASA 2012 (one RCT) (18) compared to the control group (**Table 2**).

### 3.5 Discontinuation rate and adverse events

**Table 3** summarizes the discontinuation rates for any reason and adverse events. All the included RCTs (8, 17–20) reported a discontinuation rate for any reason. The psilocybin group had discontinuation rates ranging from 2–13%, while the control group had discontinuation rates ranging from 4–21%. Three RCTs (60%, 3/5) (8, 18, 19) reported adverse events in both groups. Four RCTs reported adverse events in detail (80%) (8, 18–20). Headache was the most common adverse event in both groups (**Table 4**).

**Table 3:** Psilocybin for depression: discontinuation rate and any adverse events.

Studies	Discontinuation rate (n, %)	Psilocybin group (n, %)		Control group (n, %)	Findings^a^
Carhart-Harris et al., 2021 (UK)	8 (14)	3 (10)		5 (17)	NR
Davis et al., 2021 (USA)	3 (11)	2 (13)		1 (8)	NR
Goodwin et al., 2022 (Multicenter) (psilocybin: 25 mg vs. 1 mg)	15 (9)	5 (6)		10 (13)	NR
Goodwin et al., 2022 (Multicenter) (psilocybin: 10 mg vs. 1 mg)	19 (12)	9 (12)		10 (13)	NR
Raison et al., 2023 (USA)	12 (12)	1 (2)		11 (21)	NR
von Rotz et al., 2023 (Switzerland)	3 (6)	2 (8)		1 (4)	NR
Studies	Any adverse events (n, %)	Psilocybin group (n, %)		Control group (n, %)	Findings^a^
Carhart-Harris et al., 2021 (UK)	50 (85)	26 (87)		24 (83)	NS
Davis et al., 2021 (USA)	NR	NR		NR	NA
Raison et al., 2023 (USA)	77 (74)	44 (88)		33 (61)	P < 0.05
von Rotz et al., 2023 (Switzerland)	NR	NR		NR	NA
Goodwin et al., 2022 (Multicenter)	Any adverse events (n, %)	Psilocybin group (n, %)	Psilocybin group (n, %)	Control group (n, %)	Findings^a^
		25 mg	10 mg		
	115 (49)	44 (56)	36 (48)	35 (44)	NR

a The differences between psilocybin groups and control groups at the treatment endpoints.

NA, not applicable; NR, not reported; NS, not significant.

**Table 4:** Adverse events.

Studies	Adverse events	Psilocybin group (n, %)		Control group (n, %)	Findings^a^
Carhart-Harris et al., 2021 (UK)	Anxiety	0		4 (14)	P < 0.05
	Dry mouth	0		4 (14)	P < 0.05
	Diarrhea	1 (3)		2 (7)	NS
	Fatigue	2 (7)		7 (24)	NS
	Feeling abnormal	0		3 (10)	NS
	Feeling jittery	2 (7)		1 (3)	NS
	Headache	20 (67)		15 (52)	NS
	Migraine	3 (10)		1 (3)	NS
	Nausea	8 (27)		9 (31)	NS
	Palpitations	1 (3)		3 (10)	NS
	Sleep disorder	1 (3)		3 (10)	NS
	Vomiting	2 (7)		1 (3)	NS
Davis et al., 2021 (USA)	Adverse events	NR		NR	NA
Raison et al., 2023 (USA)	Headache	33 (66)		13 (24)	P < 0.05
	Nausea	24 (48)		3 (6)	P < 0.05
	Visual perceptual effects on dosing day	22 (44)		3 (6)	P < 0.05
	Visual perceptual effects after dosing day	3 (6)		NR	NA
von Rotz et al., 2023 (Switzerland)	Common cold	0		2 (8)	NR
	Cystitis	0		1 (4)	NR
	Diarrhea	1 (4)		0	NR
	Dizziness	2 (8)		0	NR
	Headache	4 (15)		0	NR
	Nausea	1 (4)		0	NR
Goodwin et al., 2022 (Multicenter)	Adverse events	Psilocybin (n, %)		Control group (n, %)	Findings^a^
		25 mg	10 mg		
	Anxiety	4 (5)	6 (8)	3 (4)	NR
	Depression	3 (4)	3 (4)	4 (5)	NR
	Fatigue	6 (8)	2 (3)	3 (4)	NR
	Headache	9 (11)	5 (7)	9 (11)	NR
	Insomnia	4 (5)	5 (7)	8 (10)	NR
	Mood altered	4 (5)	0	1 (1)	NR
	Suicidal ideation	5 (6)	4 (5)	2 (3)	NR

a The differences between psilocybin groups and control groups at the treatment endpoints.

NA, not applicable; NR, not reported; NS, not significant.

## 4 Discussion

To the best of our knowledge, this systematic review of five RCTs (8, 17–20) is the first to investigate the efficacy and safety of psilocybin in 472 physically healthy adults with MDD. The main findings of this systematic review were as follows: (1) 60% of the included RCTs found that psilocybin was more effective than the control group in treating depressive symptoms. However, the remaining two RCTs found inconsistent results; (2) patients with MDD treated with psilocybin showed significant improvement in anxiety symptoms compared to the control group; (3) the effectiveness of psilocybin over the control group in reducing suicidal ideation was only found in one RCT (8); and (4) the rates of any adverse events and discontinuation due to any reason were similar in both groups. The most frequent adverse event was headache in both groups.

While two RCTs (40%) (8, 18) reported mixed results, the other RCTs (60%) (17, 19, 20) found that psilocybin was significantly more effective than the placebo in improving depressive symptoms. The inconsistent results of psilocybin in the treatment of MDD can be attributed to the methodological heterogeneity of the included studies. The studies included different doses of psilocybin (1–25 mg or 0.215 mg/kg) and psychological support with different components. It is therefore difficult to distinguish between the effects of psilocybin alone and those arising from psychological support in this systematic review. Furthermore, participants across RCTs demonstrate heterogeneity in this systematic review. For example, Goodwin et al. exclusively enrolled patients with treatment-resistant depression (TRD) (18), while another study by Raison et al. (19) reported that 12.5% of participants had TRD. individuals with TRD have been associated with heightened severity and prolonged duration of illness, increased disability, and an elevated risk of suicide (55). Therefore, the proportion of patients with TRD was reported in only 3 RCTs (60%, 3/5), making it difficult to compare the efficacy and safety of psilocybin in patients with TRD with that in non-TRD patients. Patients with TRD were associated with smaller hippocampal volume compared to non-TRD patients (56). It is therefore justified to investigate the comparative efficacy and safety of psilocybin in patients with TRD versus non-TRD in the future.

Evidence exists that psilocybin-assisted therapy has substantial antidepressant effects for at least 12 months after acute intervention (57). For example, an open-label clinical trial found that marked reductions in depressive symptoms were observed after just two psilocybin treatment sessions and remained significant six-month after treatment in patients with TRD (58). Similar to ketamine and esketamine at sub-anesthetic doses (7), psilocybin has a rapid onset of antidepressant effects in MDD (20). For example, Von Rotz et al. reported a response rate of 69% 48 h after treatment (20), which was similar to the response rate of 71% for ketamine 24 h after treatment (59). A systematic review found that depressive symptoms can be rapidly and permanently reduced with the use of esketamine and psilocybin (11). However, it is unclear whether psilocybin has more rapid and sustained antidepressant effects compared to ketamine/esketamine. Interestingly, 5-methoxy-N,N-dimethyltryptamine (5-MeO-DMT), a naturally occurring tryptamine used for spiritual and recreational purposes, can alleviate depression and anxiety subjectively (60). Future RCTs should investigate the efficacy and safety of 5-MeO-DMT in patients with MDD or anxiety.

How psilocybin mediates its antidepressant and psychedelic effects is currently unknown (15). The antidepressant effects of psilocybin are believed to be caused by changes to the serotonergic system, particularly through activation of 5-HT_2A_ receptors and subsequent changes in gene expression (15). Moreover, psilocybin has the potential to indirectly modulate the dopaminergic and glutamatergic systems, as well as interact with various low-affinity receptors (15). A functional magnetic resonance imaging (fMRI) study indicated that psilocybin therapy has a distinct antidepressant mechanism, revealing that higher-order functional networks, which are rich in 5-HT_2A_ receptors, exhibited increased functional connectivity and flexibility following psilocybin treatment, whereas no such effects were observed with escitalopram (61). Psilocybin affects not only neurochemical systems, but also neural circuitry and critical brain regions associated with MDD, such as the amygdala and the default mode network.

In this systematic review, 80% of the included RCTs assessed the anxiolytic effects of psilocybin in treating MDD and consistently found that psilocybin was significantly more effective than the control in improving anxiety symptoms, which supports the results of a recent meta-analysis (16). Anxious depression is common (approximately 45.1-81.0%) in MDD (62). Anxious depression is associated with worse treatment outcomes, lower quality of life, and lower well-being than non-anxious depression (63). Interestingly, in contrast to non-anxious depressed patients, anxious depressed patients showed weaker antianhedonic (62) and antidepressant responses (64) to ketamine. Similarly, a recent study found that esketamine had a greater anti-suicidal effect on adolescents with non-anxious MDD than on those with anxious MDD (65). However, it is unclear whether psilocybin is more effective or safer for patients with anxious or non-anxious depression. Although all included RCTs assessed the anti-suicidal effects of psilocybin in the treatment of MDD, only 20% of the included studies found that psilocybin was more effective than the control group in improving suicidal ideation. Taken together, psilocybin did not appear to be effective in reducing suicidal ideation.

The most common adverse effect of psilocybin in patients with MDD was headache, although it was generally well tolerated with mild to moderate adverse events. Furthermore, adverse events related to psilocybin are generally limited to the acute dosing period (19). A recent meta-analysis revealed that a headache, the most prevalent adverse event associated with psilocybin use, is transient and does not have lasting effects (27). Notably, this meta-analysis identified significant dose-response relationships for various side effects of psilocybin, such as nausea and headache (27). Additionally, psilocybin produces acute perceptual and subjective effects in healthy volunteers (42) and is effective and safe for treating depressive symptoms in patients with other diagnoses, such as life-threatening cancer (39). A previous RCT found that psilocybin may reduce depression and anxiety in cancer patients with life-threatening diagnoses and symptoms of depression and/or anxiety (39). Overall, psilocybin is safe and well-tolerated. Studies should focus on determining the optimal dose of psilocybin to reduce depression scores while minimizing side effects.

This systematic review has several limitations. First, although we conducted a comprehensive systematic search, the number of included studies and the sample size were relatively small for qualitative synthesis. Second, a meta-analysis could not be performed because of the significant heterogeneity among the included RCTs. Third, psilocybin was usually administered with psychological support in the included studies, making it difficult to assess its isolated effects in treating MDD. Finally, the five RCTs focused on psilocybin for adult MDD, limiting the generalizability of the results to depression in other age groups.

## 5 Conclusion

Psilocybin was effective in improving depressive symptoms in over half of the included studies and reduced anxiety symptoms in patients with MDD. Further large-scale RCTs should investigate the long-term efficacy, safety, and tolerability of psilocybin for MDD.

## Data Availability

The raw data supporting the conclusions of this article will be made available by the authors, without undue reservation.

## Supplementary material

Supplementary Table 1 The PRISMA Checklist. *From:* Moher D, Liberati A, Tetzlaff J, Altman DG, The PRISMA Group (2009). Preferred Reporting Items for Systematic Reviews and Meta-Analyses: The PRISMA Statement. PLoS Med 6(6): e1000097. doi:10.1371/journal.pmed1000097. For more information, visit: www.prisma-statement.org.
